# The Anti-VEGF Epidemic: What are the Implications for Glaucoma Services?

**DOI:** 10.5005/jp-journals-10008-1106

**Published:** 2012-08-16

**Authors:** Simon E Skalicky, Colin I Clement

**Affiliations:** 1 Sydney Eye Hospital, Sydney, Australia; 2 Department of Glaucoma Unit, Sydney Eye Hospital, NSW, Australia

**Keywords:** Bevacizumab, Glaucoma, VEGF.

## Abstract

The early reports on intraocular bevacizumab injections talked about a transient predictable probably volume-related rise in intraocular pressure (IOP). As the usage of the drug increased across the globe, sustained IOP elevation was noted. This article provides an insightly into the causes of IOP spikes and its impact on glaucoma practice.

**How to cite this article:**

Skalicky SE, I Clement C. The Anti-VEGF Epidemic: What are the Implications for Glaucoma Services? J Current Glau Prac 2012;6(2):55-57.

## INTRODUCTION

Antivascular endothelial growth factor (VEGF) agents have heralded a new age of molecular therapy in ophthalmic care. They have been widely used for a range of retinal conditions and are currently investigated as treatment for corneal neovascularization and as an adjunct to trabeculectomy.^1-4^ Both their success and their rapid uptake worldwide have been remarkable.

A sustained intraocular pressue elevation (SIPE) after intravitreal injections of bevacizumab^5,6^ and ranibizumab^7,8^ is increasingly being seen. The incidence is uncertain however is probably low; several retrospective studies have been conducted recently. Good et al detected a sustained elevation in 13 of 215 eyes (6%) treated with bevacizumab or ranibizumab for exudative age-related macular degeneration (AMD).^9^ In another study by Adelman et al, four of 115 patients (3.5%) treated with bevacizumab or ranibizumab for exudative AMD developed SIPE.^10^ Mathalone et al found that 22 of 201 eyes (11%) treated with bevacizumab for exudative AMD developed this complication.^11^ Hoang et al also detected a prevalence of 11.6% in 207 patients treated with bevacizumab or ranibizumab for exudative AMD.^12^ Preexisting glaucoma, frequency of injections and cumulative number of injections have all been found to be risk factors but none consistently in these studies.^9-12^

Although these intraocular pressure (IOP) spikes are uncommon, they are often profound and unresponsive to medical therapy.^8^ Injections are offered to patients who would have likely been excluded from the initial clinical trials and for periods longer than the trials were conducted;^1,2^ it is unsurprising that there are clinical consequences not initially seen in the primary studies. The mechanism is incompletely understood and may be multifactorial. One proposed theory is a physical blockage of the trabecular meshwork (TM) by the antibody (bevacizumab) or antibody fragment (ranibizumab); alternatively *in vitro* data suggests that the anti-VEGF agents may be directly toxic to TM endothelial cells.^13^ Another theory is that nondegradeable silicone particles from the syringe or stopper obstruct the TM.^14^ Mishandling of the medication in storage and freeze-thawing may lead to particles leeching into solution.^15^ Particulate contamination may account for unilateral cases in individuals receiving bilateral injections. Eyes receiving multiple injections are likely to be at increased risk as are those with preexisting TM dysfunction.^8,9^

Are anti-VEGF injections increasing glaucoma workload? Intravitreal ranibizumab was approved for the treatment of exudative AMD in 2006 in the USA and in 2007 in Europe, UK, Australia and Canada. Since then, the number of intravitreal injections performed has risen steadily in these regions.

**Figs 1A to C:**
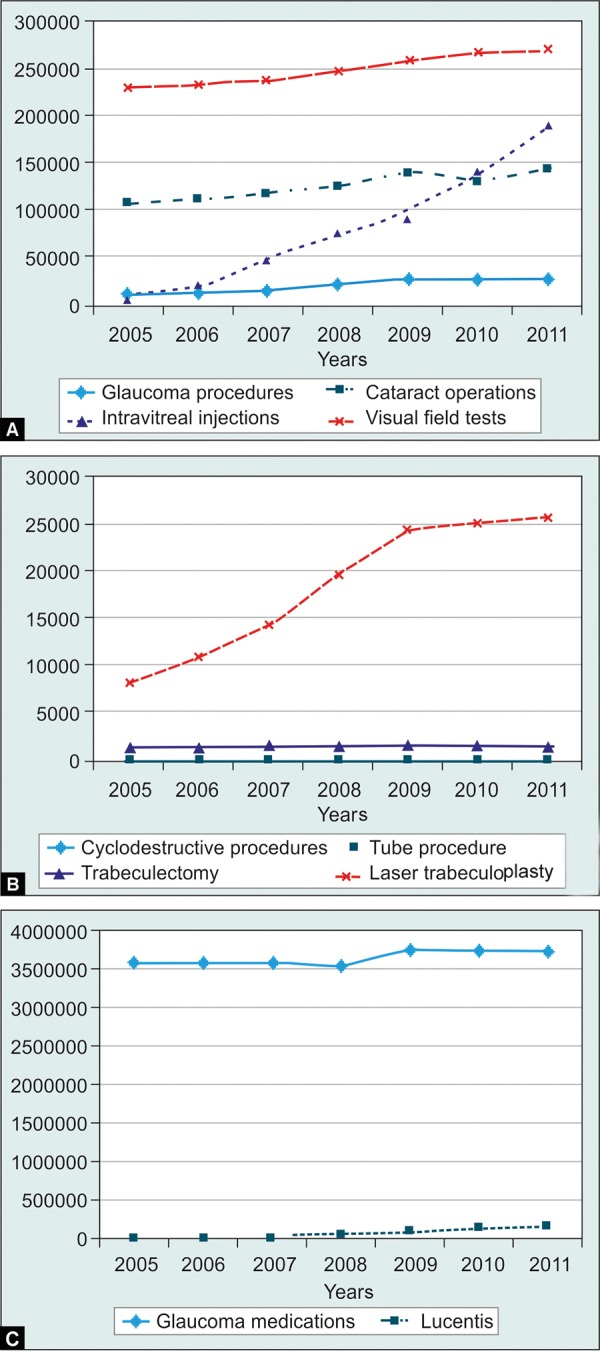
Medicare benefits schedule and pharmaceutical benefits schedule (Australia) data, (A) glaucoma procedures, cataract operations, intravitreal injections and computerized field tests, (B) glaucoma procedures subdivided, (C) dispensation of pharmaceuticals: Topical glaucoma medications and lucentis

**Figs 2A and B:**
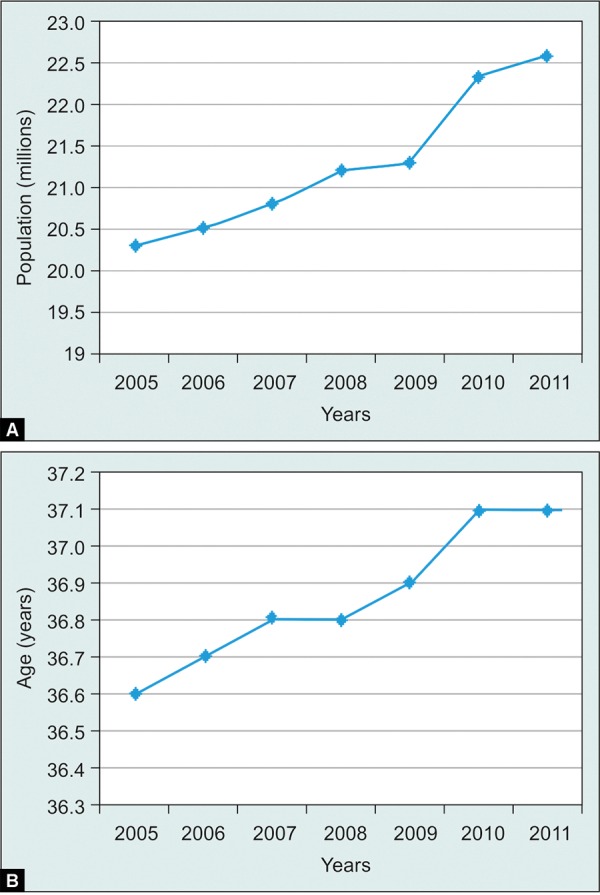
Australian population trends: (A) Population, (B) median age

A review of Australian Medical Benefits Schedule (AMBS) data from 2005 to 2011 demonstrates a dramatic rise in intravitreal injections from 6,867 procedures in 2005 to 189,759 in 2011 (Fig. 1A).^16^ Using a prevalence of 3.5% to 11.6% based on retrospective data,^9-12^ one would predict a large volume of glaucoma work generated by anti-VEGF injections. However, the AMBS data does not demonstrate this. Over this 7 years period, there has been a modest rise in glaucoma procedures and computerized visual field testing, however, this rate is similar to the increase in cataract surgery and may reflect natural population growth and ageing (Figs 2A and B).^17^ During this period in Australia there has been a rapid uptake of computerized visual field machines by optometrists, and there has been a similar uptake of selective laser trabeculoplasty (SLT) by ophthalmologists. When glaucoma procedures are subdivided into procedure types only SLT has increased; glaucoma surgical numbers have not changed (Fig. 1B). Data from the Australian Pharmaceutical Benefits Scheme (APBS) regarding ranibizumab and all topical glaucoma medications dispensed to Australians from 2005 to 2011 shows little change in glaucoma medications during the rise of ranibizumab usage (Fig. 1C).^18^ This does not support a significant increase in glaucoma during this period.

How do we explain the discrepency between AMBS and APBS data and the predicted incidence of anti-VEGF-related SIPE? Perhaps SIPE is less common than current studies predict; certainly there is a need for larger prospective studies. Perhaps it is occuring in patients who would have developed glaucoma even without receiving the injections, although the rapid and dramatic pattern of IOP rise post injection implies a direct causative role of the anti-VEGF treatment. The most likely reason is that the volume of glaucoma treatment dwarfs exudative AMD treatment (see Fig. 1C); these IOP spikes are not common enough to significantly alter the volume of glaucoma work on a national level. However, this may change; in 2010 ranibizumab was approved in USA for the treatment of macula edema secondary to vein occlusions^19^ and diabetic retinopathy^20^ and may soon be routinely used for these indications in Australia; already many patients are receiving bevacizumab (off label). Compared with AMD patients these patients are being treated at an earlier stage of life and for potentially longer periods. One would expect the use of intravitreal anti-VEGF agents to continue to rise―we may soon see a corresponding rise in glaucoma workload.

If anti-VEGF agents are not yet changing the volume of glaucoma practice, are they changing the nature of glaucoma practice? Certainly, they have been useful in managing neovascular glaucoma; if administered early bevacizumab prevents an intractable pressure rise,^21^ however, does not replace conventional medical, laser and surgical intervention in advanced cases.^22^ As an adjunct to trabeculectomy bevacizumab has not been shown to significantly effect the final IOP, however, larger studies are required to further evaluate its role.^3,23,24^

Anti-VEGF agents induce SIPE in a minority of patients receiving intravitreal injections. Although the glaucoma workload in Australia has not risen significantly due to this, it may increase soon owning to the projected increase in anti-VEGF injections. Newer longer-acting anti-VEGF agents are likely to be introduced into mainstream practice;^25,26^ it is unclear how these will effect the incidence of SIPE.
